# Molecular Mechanisms and Tumor Biological Aspects of 5-Fluorouracil Resistance in HCT116 Human Colorectal Cancer Cells

**DOI:** 10.3390/ijms22062916

**Published:** 2021-03-13

**Authors:** Chinatsu Kurasaka, Yoko Ogino, Akira Sato

**Affiliations:** 1 Department of Biochemistry and Molecular Biology, Faculty of Pharmaceutical Sciences, Tokyo University of Science, 2641 Yamazaki, Noda, Chiba 278-8510, Japan; 3a16042@ed.tus.ac.jp (C.K.); ogino@rs.tus.ac.jp (Y.O.); 2 Department of Gene Regulation, Faculty of Pharmaceutical Sciences, Tokyo University of Science, 2641 Yamazaki, Noda, Chiba 278-8510, Japan

**Keywords:** colorectal cancer cells, drug resistance, 5-Fluorouracil, thymidylate synthase, exome sequencing

## Abstract

5-Fluorouracil (5-FU) is a cornerstone drug used in the treatment of colorectal cancer (CRC). However, the development of resistance to 5-FU and its analogs remain an unsolved problem in CRC treatment. In this study, we investigated the molecular mechanisms and tumor biological aspects of 5-FU resistance in CRC HCT116 cells. We established an acquired 5-FU-resistant cell line, HCT116R^F10^. HCT116R^F10^ cells were cross-resistant to the 5-FU analog, fluorodeoxyuridine. In contrast, HCT116R^F10^ cells were collaterally sensitive to SN-38 and CDDP compared with the parental HCT16 cells. Whole-exome sequencing revealed that a cluster of genes associated with the 5-FU metabolic pathway were not significantly mutated in HCT116 or HCT116R^F10^ cells. Interestingly, HCT116R^F10^ cells were regulated by the function of thymidylate synthase (TS), a 5-FU active metabolite 5-fluorodeoxyuridine monophosphate (FdUMP) inhibiting enzyme. Half of the TS was in an active form, whereas the other half was in an inactive form. This finding indicates that 5-FU-resistant cells exhibited increased TS expression, and the TS enzyme is used to trap FdUMP, resulting in resistance to 5-FU and its analogs.

## 1. Introduction

Colorectal cancer (CRC) is the third-most common cancer in the world [1], and 5-Fluorouracil (5-FU) is the most important chemotherapeutic agent used in its treatment [2,3]. 5-FU is also widely used to treat other cancers, such as gastric, pancreatic, breast, ovarian, and head and neck cancers [2,3]. 5-FU is converted to 5-fluorodeoxyuridine monophosphate (FdUMP), which is a potent inhibitor of thymidylate synthase (TS) [3,4,5]. FdUMP forms a covalent complex with TS in the presence of 5,10-methylenetetrahydrofolate (CH_2_–THF) [2,3,5]. The inhibition of TS depletes the intracellular dTTP pool and subsequently inhibits DNA synthesis [2,3,4,5]. Another effect by which 5-FU can exert its cytotoxic action is its incorporation as fluorodeoxyuridine triphosphate (FdUTP) and fluorouridine triphosphate (FUTP) into DNA and RNA, respectively [2,3,4]. Experimental and clinical studies indicate that continuous exposure of CRC cells to 5-FU results in acquired resistance to 5-FU and its derivatives. This is often caused by common cancer resistance mechanisms, such as drug inactivation, drug efflux, drug target alterations, bypass pathway activation, DNA damage repair, and cell death [2,3]. 5-FU resistance is correlated with the level of TS protein and enzymatic activity in cancer cells [2,3,6,7,8]. In addition, high TS protein and RNA expression levels in tumor tissue is also a useful biomarker for poor prognosis for 5-FU-based chemotherapy in CRC patients [2,3,9]. Furthermore, 5-FU sensitivity is influenced by the expression levels of dihydropyrimidine dehydrogenase (DPD) [9,10], which converts 5-FU to dihydrofluorouracil (DHFU) during the catabolic process [2,3,4,9,10]. However, 5-FU resistance has not yet been circumvented clinically.

In this study, we established a 5-FU-resistant HCT116 CRC cell line (HCT116R^F3^ and HCT116R^F10^) and analyzed its biological features. HCT116R^F10^ cells, which are cross-resistant to the 5-FU analog fluorodeoxyuridine (FUdR), were collaterally sensitive to SN-38 and CDDP compared with the parental HCT16 cells. In addition, HCT116R^F10^ cells exhibited a lower ability to form tumor spheres compared with parental HCT116 cells. Notably, HCT116R^F10^ cells maintained the tumor sphere formation ability compared with HCT116 cells under 5-FU exposure conditions. Furthermore, a gene cluster associated with 5-FU metabolic pathway was not significantly mutated in HCT116 and HCT116R^F10^ cells as determined by whole-exome sequencing. We found that HCT116R^F10^ cells regulate intracellular TS states in which half of the TS enzyme is in a functional form and the other half exists as an FdUMP-covalent complex (inactive form). These findings provide a better understanding of resistance to anticancer 5-FU and its analogs.

## 2. Results

### 2.1. Establishment of the 5-Fluorouracil-Resistant HCT116 Cells

**Figure 1:**
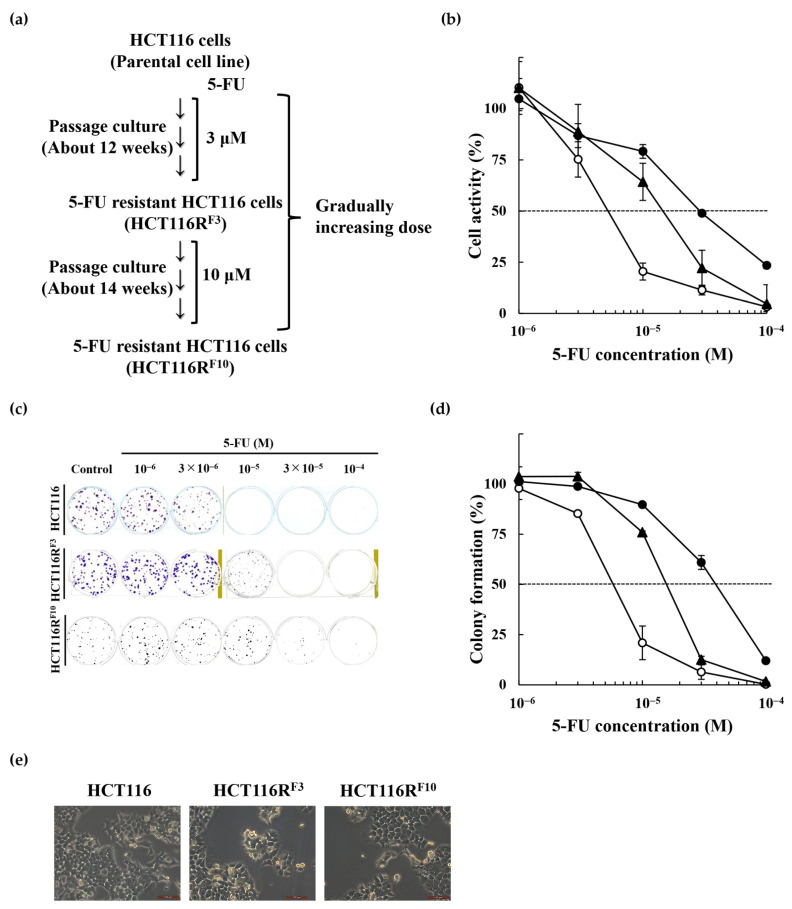
Establishment of HCT116R^F10^, a 5-FU-resistant derivative of the human colorectal cancer cell line HCT116. (**a**) Scheme for the establishment of the 5-FU-resistant HCT116 cells (HCT116R^F10^). (**b**) HCT116R^F10^ and parental HCT116 cells were tested for cell viability after a 72 h treatment with 5-FU. Results represent the averages of three independent experiments with error bars showing ±SE from triplicates. (**c**) Drug sensitivities of HCT116R^F10^ and HCT116 using the colony formation assay. HCT116R^F10^ and HCT116 cells were treated with the indicated concentration of 5-FU and incubated for 10 days. (**d**) HCT116 cells: Colony formation (%) represents the average of two independent experiments, each performed in duplicate, with error bars showing ±SE of four measurement values. HCT116R^F3^ and R^F10^ cells: Colony formation (%) represents the average of three independent experiments, each performed in triplicate, with error bars showing ±SE of nine measurement values. White circle, HCT116 cells; black triangle, HCT116R^F3^ cells; black circle, HCT116R^F10^ cells. (**e**) Morphological features were analyzed using a Leica DMi1 microscope with LAS V4.12 at 200× magnification. Scale bar = 100 μm.

To elucidate the mechanisms underlying resistance to 5-FU, we generated a variant of the HCT116 human colorectal cancer cell line that was resistant to 5-FU, an important anticancer drug used for CRC treatment [2,3]. We established 5-FU-resistant HCT116R^F3^ or HCT116R^F10^ cells by repeated exposure of parental HCT116 cells to stepwise increasing concentrations of 5-FU over a period of approximately 12 weeks at 3 μM and 14 weeks at 10 μM, respectively (Figure 1a). The EC_50_ of 5-FU in HCT116R^F3^ (intermediate variant) and HCT116R^F10^ cells were determined by a WST-8 assay after continuous exposure for 72 h. As shown in Table 1 and Figure 1b, the EC_50_ value of the 5-FU-resistant HCT11 6 cells was higher (1.5 × 10^−5^ M in HCT116R^F3^ and 2.9 × 10^−5^ M in HCT116R^F10^ cells) than that of sensitive, parental HCT116 cells (5.1 × 10^−6^ M). The RI was approximately 2.9 for HCT116R^F3^ cells and 5.7 for HCT116R^F10^ cells (Table 1). In addition, similar results were obtained by colony formation assay (Figure 1c,d). The EC_50_ value of 5-FU-resistant HCT116 cells was significantly higher (1.6 × 10^−5^ M in HCT116R^F3^ and 3.8 × 10^−5^ M in HCT116R^F10^ cells) than that of the parental HCT116 cells (5.5 × 10^−6^ M) (Table 1). The RI of HCT116R^F3^ and HCT116R^F10^ cells was approximately 2.9 and 6.9, respectively (Table 1). Furthermore, parental HCT116, HCT116R^F3^, and HCT116R^F10^ cells exhibited nearly similar morphological features (Figure 1e).

### 2.2. Anticancer Drug Response of the 5-FU-Resistant HCT116 Cells

**Figure 2:**
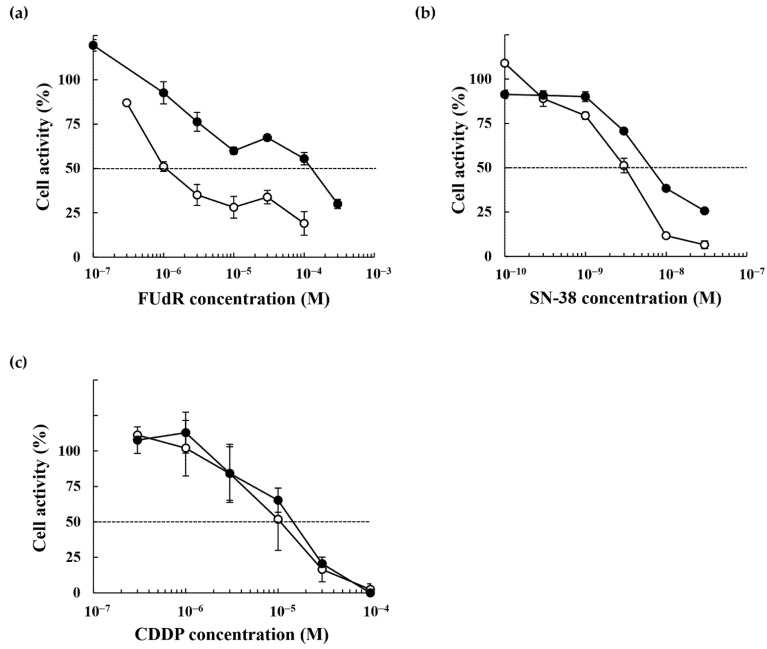
Sensitivity of 5-FU-resistant HCT116R^F10^ and parental HCT116 cells to FUdR, SN-38, and CDDP. The cell proliferation WST-8 assay of HCT116R^F10^ and parental HCT116 cells after a 72 h treatment with (**a**) FUdR, (**b**) SN-38, and (**c**) CDDP. Results represent the averages of two independent experiments, with error bars showing ±SE of triplicates. White circle, HCT116 cells; black circle, HCT116R^F10^ cells.

**Figure 3:**
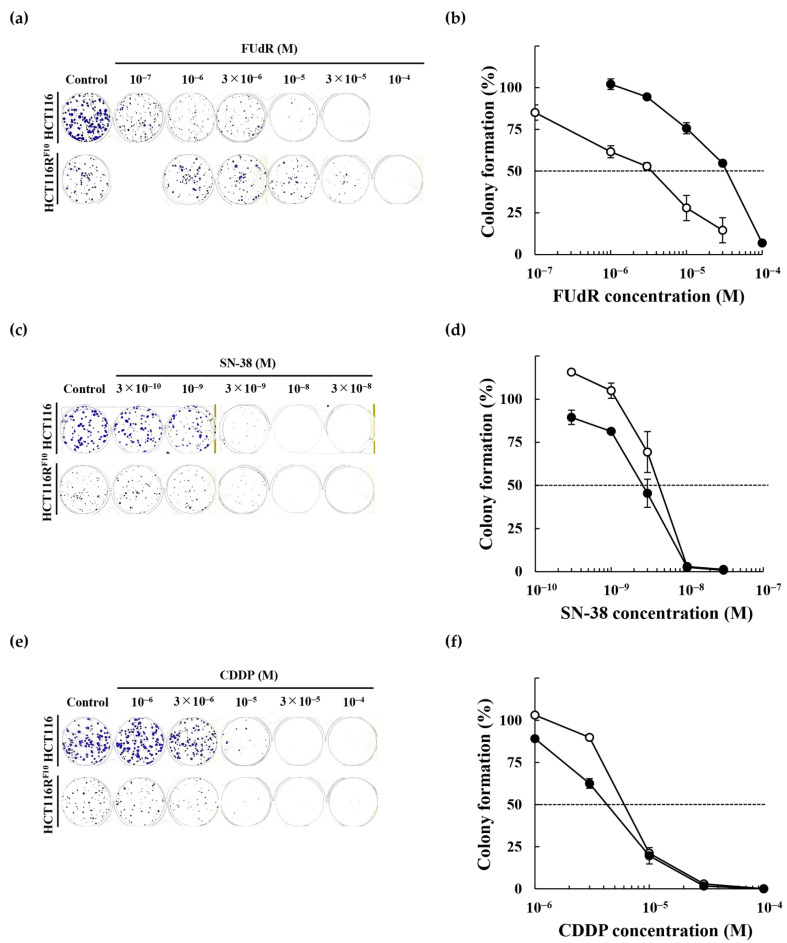
Sensitivity of 5-FU-resistant HCT116R^F10^ and parental HCT116 cells to FUdR, SN-38, and CDDP. Colony formation by HCT116R^F10^ and parental HCT116 cells after 10 days of treatment with (**a**,**b**) FUdR, (**c**,**d**) SN-38, and (**e**,**f**) CDDP. Colony formation (%) represents the averages of two independent experiments each performed in duplicate (**b**) or triplicate (**d**,**f**), with error bars showing ±SE of four (**b**) or six (**d**,**f**) measurement values. White circle, HCT116 cells; black circle, HCT116R^F10^ cells.

We examined the effects of several anticancer drugs, including the 5-FU analog FUdR, SN-38, an active metabolite of irinotecan, and CDDP, on the proliferation of parental HCT116 and 5-FU-resistant HCT116R^F10^ cells by WST-8 (Figure 2) and colony formation assays (Figure 3). As shown in Table 2 and Figure 2a, HCT116R^F10^ cells were 80.0-fold (EC_50_ = 1.2 × 10^−4^ M) more resistant to FUdR than parental HCT116 cells (EC_50_ = 1.5 × 10^−6^ M). In contrast, the resistant index for SN-38 and CDDP was 2.1-fold (EC_50_ = 6.6 × 10^−9^ M in HCT116R^F10^ cells; 3.1 × 10^−9^ M in HCT116 cells) and 1.4-fold (EC_50_ = 1.4 × 10^−5^ M in HCT116R^F10^ cells; 1.0 × 10^−5^ M in HCT116 cells), respectively (Table 2, Figure 2b,c). Similarly, for the colony-forming assay, HCT116R^F10^ cells were 9.7-fold (EC_50_ = 3.3 × 10^−5^ M) more resistant to FUdR than the parental HCT116 cells (EC_50_ = 3.4 × 10^−6^ M) (Figure 3a). In addition, the RI of SN-38 and CDDP was 0.7-fold (EC_50_ = 3.0 × 10^−9^ M in HCT116R^F10^ cells; 4.2 × 10^−9^ M in HCT116 cells) and 0.9-fold (EC_50_ = 4.5 × 10^−6^ M in HCT116R^F10^ cells; 5.2 × 10^−6^ M in HCT116 cells), respectively (Table 2, Figure 3b,c). These results indicate that 5-FU-resistant HCT116R^F10^ cells exhibit cross-resistance to FUdR but collateral sensitivity to the anticancer drugs SN-38 and CDDP. This finding suggests that the HCT116R^F10^ cells are resistant not only to 5-FU but also to other 5-FU deoxyribose analogs such as FUdR.

### 2.3. Biological Features of the 5-FU-Resistant HCT116 Cells

**Figure 4:**
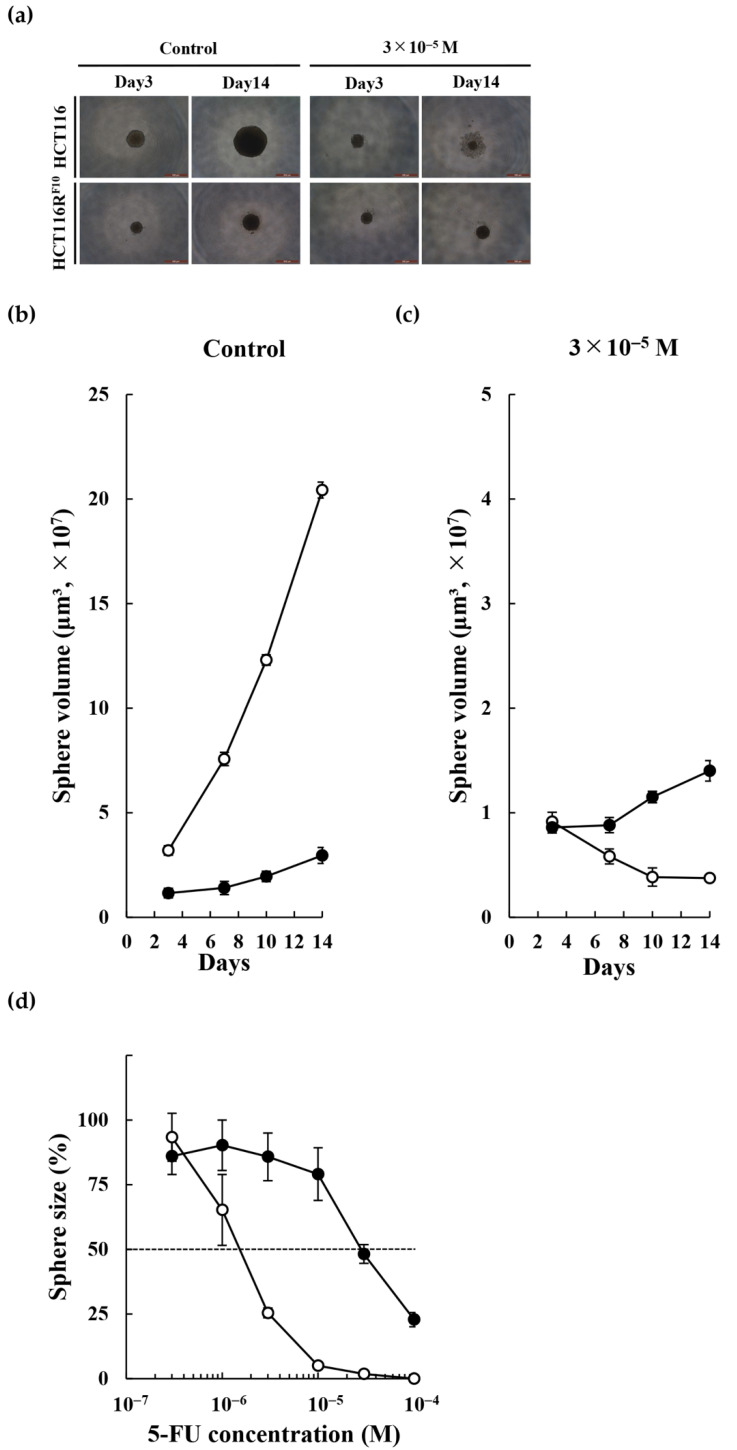
Tumor sphere formation of 5-FU-resistant HCT116R^F10^ and parental HCT116 cells. (**a**) Tumor sphere formation was analyzed using a Leica DMi1 microscope with 50× magnification. Scale bar = 500 μm. HCT116R^F10^ and parental HCT116 cells were treated with or without 3 × 10^−5^ M 5-FU for 3 or 14 days. Control, no 5-FU, solvent (DMSO) alone. To assess the ability of HCT116R^F10^ and parental HCT116 cells to form tumor spheres, the cells were treated with solvent alone (**b**) or 3 × 10^−5^ M 5-FU (**c**) for 14 days. Tumor sphere size was calculated as described in the Materials and Methods. White circle, HCT116 cells; black circle, HCT116R^F10^ cells. (**d**) Drug sensitivity of 5-FU in HCT116 and HCT116R^F10^ tumor spheres. Tumor sphere formation by HCT116R^F10^ and parental HCT116 cells after a 14-day treatment with 5-FU at the indicated concentrations. Results are the averages for groups of three tumor spheres each with error bars showing SE. White circle, HCT116 cells; black circle, HCT116R^F10^ cells.

We analyzed the tumor sphere formation ability of HCT116R^F10^ cells and parental HCT116 cells in three-dimensional cell culture experiments (Figure 4). HCT116R^F10^ cells exhibited a lower ability to form tumor spheres compared with parental HCT116 cells under untreated conditions (Figure 4a left panel and b). Interestingly, HCT116R^F10^ cells maintained a tumor sphere formation ability compared with parent HCT116 cells during 5-FU treatment conditions (Figure 4a,c). We next examined the sensitivity of parental HCT116 and HCT116R^F10^ tumor sphere cells to 5-FU. As shown in Figure 4d, HCT116R^F10^ cells were 18.7-fold (EC_50_ = 2.8 × 10^−5^ M) more resistant to 5-FU than parental HCT116 cells (EC_50_ = 1.5 × 10^−6^ M). These data indicate that 5-FU-resistant HCT116R^F10^ cells are less prone to tumorigenesis than sensitive, parental HCT116 cells, but formed tumor spheres that retained a higher 5-FU resistance.

### 2.4. Exome Sequencing Analysis of HCT116 Parent Cells and 5-FU-Resistant HCT116R^F10^ Cells

**Figure 5:**
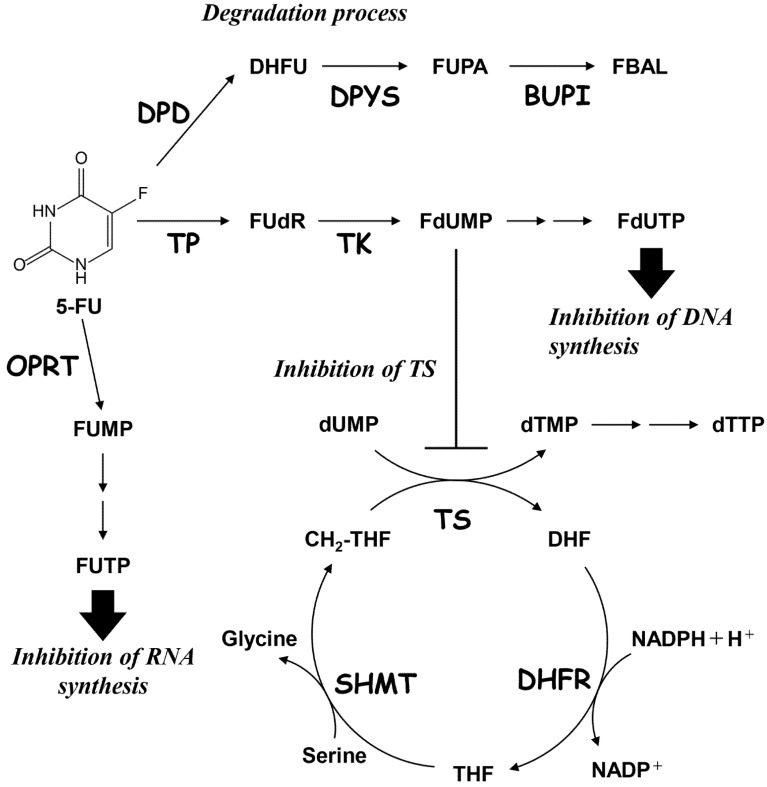
Metabolic pathways associated with 5-FU. 5-FU, 5-fluorouracil; DHFU, dihydrofluorouracil; FUPA, fluoroureidopropionate; FBAL, fluoroalanine; FUdR, fluorodeoxyuridine; FdUMP, fluorodeoxyuridine monophosphate; FdUTP, fluorodeoxyuridine triphosphate; FUMP, fluorouridine monophosphate; FUTP, fluorouridine triphosphate; dUMP, deoxyuridine monophosphate; dTMP, deoxythymidine monophosphate; dTTP, deoxythymidine triphosphate; CH_2_-THF, 5,10-methylenetetrahydrofolate; DHF, dihydrofolate; THF; tetrahydrofolate; TS, thymidylate synthase; DPD, dihydropyrimidine dehydrogenase; DPYS, dihydro pyrimidase; DHFR, dihydrofolate reductase; BUPI, β-ureido propionase; TP, thymidine phosphorylase; TK, thymidine kinase; SHMT, serine hydroxymethyltransferase; and OPRT, orotate phosphoribosyltransferase 1.

We analyzed variants of 5-FU metabolic pathway-related enzyme genes, including *TYMS*, which encodes for TS, and *DPYD*, which encodes for DPD in HCT116 and HCT116R^F10^ cells. TS is a major intracellular target of 5-FU, whereas DPD catalyzes the rate-limiting step in the catabolism of 5-FU [2,3,11]. The pathways involved in the metabolism of 5-FU and its analog FUdR are shown in Figure 5. The genetic alteration status of nearly all of the 5-FU metabolic pathway-related genes was of similar status in both cells. Importantly, the variants of *TYMS* and *DPYD* in HCT116 and HCT116R^F10^ cells contained heterozygous mutations or intron variants. We identified two *TYMS* intron variants, 454+197_454+202delTTTTTT and 454+199_454+202delTTTT, in HCT116R^F10^ cells. In contrast, only one *TYMS* intron variant, 454+200_454+202delTTT, was present in sensitive parental HCT116 cells. Similarly, the three *DPYD* variants, 2999A>T, 2623-59T>G, and 2442+78delA, were present in the HCT116R^F10^ cells. In addition, three *DPYD* variants, 2442+77_2442+delAA, 40-461delT, and -113T>C, were present in HCT116 cells. Herein, we show that one of the *DPYD* heterozygous variants, 2999A>T, is a missense mutation (Asp1000Val) in DPD of 5-FU-resistant HCT116R^F10^ cells.

### 2.5. Regulation of TS and DPD in HCT116 Parent Cell and 5-FU-Resistant HCT116R^F10^ Cells

**Figure 6:**
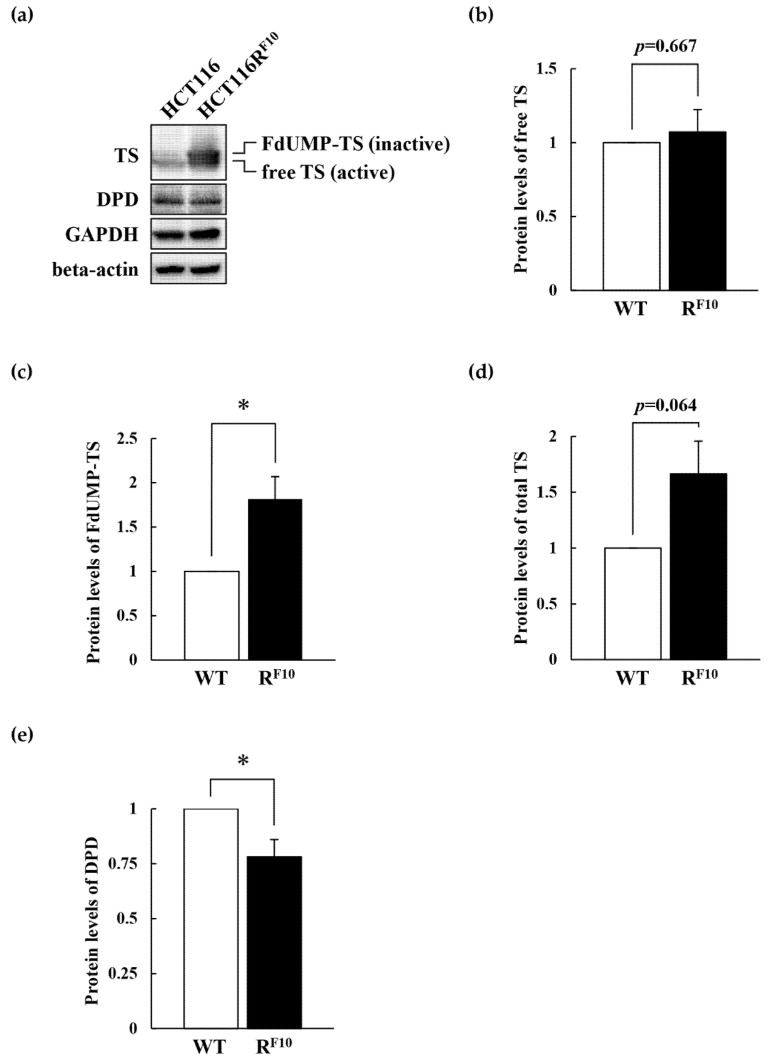
Protein levels of TS and DPD in 5-FU-resistant HCT116R^F10^ and parental HCT116 cells. (**a**) Whole-cell lysates were prepared from parental HCT116 and HCT116R^F10^ cells. Protein levels of TS, DPD, GAPDH, and beta-actin were measured by Western blot analysis. The expression levels of GAPDH and beta-actin were used as an internal control. Data are representative of at least three independent experiments. Protein levels of (**b**) free TS, (**c**) FdUMP-TS, (**d**) total TS, and (**e**) DPD in parental HCT116 and HCT116R^F10^ cells. Levels of TS and DPD protein in HCT116R^F10^ cells are represented by the ratio of TS or DPD density to GAPDH density relative to the value for parental HCT116 cells. Results represent the averages of three independent experiments with error bars showing ±SE of triplicates. * *p* < 0.05.

**Figure 7:**
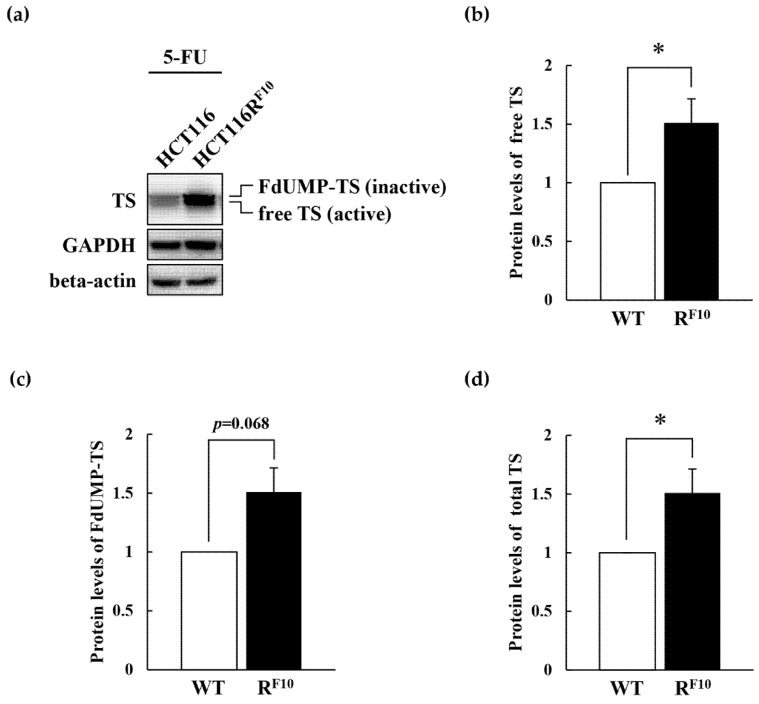
Protein levels of TS in 5-FU-resistant HCT116R^F10^ and parental HCT116 cells after treatment with 5-FU. (**a**) Whole-cell lysates were prepared from parental HCT116 and HCT116R^F10^ cells after 24 h treatment with 1 × 10^−4^ M 5-FU. Protein levels of TS, GAPDH, and beta-actin were measured by Western blot analysis. Data are representative of at least three independent experiments. Protein levels of (**b**) free TS, (**c**) FdUMP-TS, and (**d**) total TS in parental HCT116 and HCT116R^F10^ cells. Levels of TS protein in HCT116R^F10^ cells are represented by the ratio of TS density to GAPDH density relative to the value for parental HCT116 cells. Results represent the averages of three independent experiments with error bars showing ±SE of triplicates. * *p* < 0.05.

To elucidate the association of TS and DPD expression with 5-FU resistance, we analyzed TS and DPD expression levels in parental HCT116 and 5-FU-resistant HCT116R^F10^ cells by Western blot analysis (Figure 6a). Interestingly, as shown in Figure 6a (top panel) and 6b, free-TS protein levels were almost identical in HCT116R^F10^ and HCT116 cells. Conversely, the FdUMP–TS covalent complex was 1.8-fold higher in HCT116R^F10^ cells than in HCT116 cells (Figure 6a top panel and 6c). Importantly, it should be noted that total TS, the free form, the FdUMP-covalent form, and total TS was overexpressed in HCT116R^F10^ cells rather than in HCT116 cells (Figure 6a top panel and Figure 6d). The upper band of TS, indicated FdUMP-covalent form, which represents TS in ternary complexes and is correlated with the intracellular concentration of FdUMP [12,13,14]. In addition, DPD protein levels were slightly decreased in HCT116R^F10^ cells than in parental HCT116 cells (Figure 6a second panel and Figure 6e). GAPDH and beta-actin were used as an internal controls (Figure 6a third and bottom panels). In parental HCT116 cells and HCT116R^F10^ cells, both internal control proteins, GAPDH and beta-actin, had similar levels. After treatment with 1 × 10^−4^ M 5-FU for 24 h, the protein levels of free TS, FdUMP–TS covalent complex, and total TS were individually about 1.5-fold higher in HCT116R^F10^ cells than in parental HCT116 cells (Figure 7a–d). Intriguingly, these data indicated that the proportion of active free TS in the intracellular total TS was highly regulated in the 5-FU resistant HCT116R^F10^ cells. These findings suggested that the regulation of TS status, which includes the balance of active free TS or the inactive FdUMP–TS covalent complex, may confer resistance to 5-FU.

## 3. Discussion

5-FU and its derivatives are widely used in anticancer chemotherapy [2,3]. Studies to date have shown that cancer cells develop resistance to 5-FU through complex mechanisms [2,3]. Of note, the TS enzyme and other enzymes involved in 5-FU anabolism or catabolism are often altered in expression or function to promote 5-FU resistance [2,3]. In addition, altered cell death and autophagy, expression/functional changes in drug transporters, epigenetic changes, and non-coding RNA (i.e., microRNA and long non-coding RNA) dysfunction represent putative 5-FU-resistant mechanisms [2,3]. It has been widely believed that TS is the main molecular mechanism that influences 5-FU sensitivity and targeting TS is a major strategy for reversing 5-FU resistance. Importantly, there are currently no specific therapies to overcome 5-FU resistance.

We established a 5-FU-resistant cell line, HCT116R^F10^, and analyzed its characteristics. Importantly, HCT116R^F10^ cells were cross-resistant to the 5-FU analog, FUdR (Figure 2 and Figure 3). In contrast, HCT116R^F10^ cells did not exhibit cross-resistance to the anticancer drugs, SN-38 and CDDP (Figure 2 and Figure 3). Similarly, Boyer et al. also reported that 5-FU-resistant HCT116 cells were not cross-resistant to oxaliplatin or irinotecan [15]. In addition, the sensitivities to 5-FU and FUdR were similar to the levels observed individually in parental HCT116 cells. Of note, previous reports indicated that FUdR is more potent than 5-FU and that the inhibition of cell proliferation was approximately 10- to 100-fold higher than that of 5-FU in multiple cancer cell lines [16,17,18]. These findings suggest that the common target or mechanism of action of 5-FU and FUdR is the key to 5-FU resistance in this resistant cell model. Furthermore, our results revealed that HCT116R^F10^ cells are resistant to 5-FU and its derivatives, but are not multidrug resistant.

To elucidate the underlying cause of 5-FU resistance, we investigated 5-FU metabolism-related genes, including *TYMS* and *DPYD*, in HCT116R^F10^ and parent HCT116 cells by using whole-exome sequencing. The results revealed that the genetic alteration of almost all of the 5-FU metabolic pathway-related genes was similar in status, intron variants, and heterozygous mutation in both cells (Table 3). Interestingly, we found that the one functional DPD mutation, Asp1000Val, is present in HCT116R^F10^ cells. However, the effects of *DPYD* missense mutation on 5-FU resistance are not well understood.

Next, to evaluate TS and DPD in HCT116R^F10^ and parent HCT116 cells, we analyzed the expression of these genes by Western blot analysis (Figure 6). 5-FU and FUdR are converted to FdUMP, and it has been shown to form a covalent complex with TS in the presence of CH_2_-THF [2,3,5]. Our results indicated that the free-TS protein (active form) levels were similar in HCT116R^F10^ and HCT116 cells. Interestingly, the FdUMP–TS covalent complex (inactive form) was higher in HCT116R^F10^ cells than in HCT116 cells. Notably, this result indicates that TS is not overexpressed, but rather there are two types of TS in HCT116R^F10^ cells: free TS and FdUMP-coupled TS. We observed that 5-FU-resistant HCT116R^F10^ cells exhibit upregulated *TYMS* expression and use a fraction of TS to trap FdUMP, resulting in resistance to 5-FU and its analogs. In addition, our data suggest that the regulation of the TS complex, which refers to the balance of the active free-TS form and the inactive FdUMP–TS covalent complex, may confer to 5-FU resistance.

Numerous studies have shown that *TYMS* gene amplification, leading to mRNA and enzyme overproduction, is a major mechanism of resistance to fluoropyrimidines 5-FU and FUdR and their derivatives [19]. Also, free TS binds to its own mRNA, resulting in translational repression, that is, translational autoregulation [12,20,21,22,23]. Indeed, TS ligands, including 5-FU, disrupt the interaction of the TS enzyme with TS mRNA, leading to translational derepression and enzyme upregulation [12,22,23]. Additionally, to translational derepression, enzyme stabilization has been indicated as the primary mechanism of TS induction by fluoropyrimidines in human colon and ovarian cancer cell lines [24,25,26]. Furthermore, it is proposed that fluoropyrimidine-mediated increases in TS levels occur through an effect on enzyme stability with no effect on its mRNA [25,27]. It is also suggested that TS stabilization could be the result of conformational changes that may occur upon the formation of a ternary complex, reducing the susceptibility of the TS enzyme to proteolysis [28]. These findings indicated that understanding translational derepression and enzyme stabilization as the process of TS induction has significance for elucidating the mechanism of resistance acquisition. Further investigation is needed on the functions of the FdUMP–TS covalent complex and free TS in both translational regulation and enzyme stabilization for fluoropyrimidine resistance mechanisms using 5-FU-resistance and 5-FU-sensitive parental HCT116 cell lines. Collectively, our findings provide a better understanding of the anticancer drugs, 5-FU and its fluoropyrimidine derivatives, with respect to resistance mechanisms and anticancer treatment strategies.

## 4. Materials and Methods

### 4.1. Reagents

The anticancer drugs 5-FU, FUdR, CDDP, and SN-38 were obtained from FUJIFILM Wako Pure Chemical Corporation (Osaka, Japan). 5-FU, CDDP, and SN-38 were stored as 100 mM stocks in dimethyl sulfoxide (DMSO, Sigma-Aldrich; Merck KGaA, Darmstadt, Germany) at −20 °C. FUdR was stored as a 20 mM stock solution in ultrapure water at −20 °C.

### 4.2. Cell Culture

The human colon cancer cell line HCT116 was obtained from the American Type Culture Collection. Parental and 5-FU-resistant HCT116 cell lines were cultured in DMEM medium containing 10% heat-inactivated fetal bovine serum, 100 units/mL penicillin, and 100 μg/mL streptomycin in a 37 °C incubator under an atmosphere containing 5% CO_2_ and 100% relative humidity.

### 4.3. Generation of the 5-FU-Resistant HCT116 Cell Line

5-FU-resistant HCT116 cells were obtained by continuous exposure of cells to 3 μM 5-FU for approximately 12 weeks and following at 10 μM for an approximate 14-week period. A derivative of HCT116 was isolated and named HCT116R^F3^ or HCT116R^F10^. The HCT116R^F10^ cells were maintained in culture in the presence of 10 μM 5-FU.

### 4.4. Cell Viability by WST-8 Assay

Cell viability assays were performed as previously described [29]. Cell viability was determined using the WST-8 (Cell Counting Kit-8) cell proliferation assay (Dojindo, Tokyo, Japan). Briefly, cells were seeded into 96-well plates (1000 cells per well) in triplicate and then treated with various concentrations of anticancer drugs or DMSO and water (as a negative control). Following incubation for 72 h, WST-8 reagent was added to each well and the plate was placed in a 5% CO_2_ incubator at 37 °C for an additional 1 h. Optical density was measured at 450 nm on a Tecan microplate reader (Mannedorf, Switzerland). The EC_50_ value was defined as the concentration of drug producing 50% inhibition of cell proliferation. The resistance index (RI) was defined as the ratio of EC_50_ values between the resistant and parental cell lines. Experiments were repeated at least three times.

### 4.5. Colony Formation Assay

Colony formation assay was performed as previously described [29,30,31,32]. HCT116 and HCT116R^F10^ cells were dissociated with Accutase, suspended in medium, inoculated into 6-well plates (200 cells per well) in triplicate, and then incubated overnight. The cells were treated with various concentrations of drugs or with solvent (DMSO or water) as a negative control. After incubation for 10 days, cells were fixed with 4% formaldehyde solution and stained with 0.1% (*w*/*v*) crystal violet, and the number of colonies in each well was counted.

### 4.6. Tumor Sphere Assay

HCT116 and HCT116R^F10^ cells were seeded into 96-well PrimeSurface^®^ plate 96U (Sumitomo Bakelite Co., Ltd., Tokyo, Japan) plates (1000 cells per well) in triplicate and then treated with various concentrations of 5-FU or DMSO (as a negative control). Following incubation for 14 days, tumor sphere size was monitored once every 3–4 days. Tumor sphere volume (*V*) was calculated using the following formula: *V* = *ab*^2^/2 (*a* and *b* are the long and short diameters of the tumor sphere, respectively).

### 4.7. Exome Sequencing Analysis

DNA extraction was performed as previously described [29]. Genomic DNA was extracted from cells (5 × 10^6^ cells) by using a DNeasy Tissue Kit (QIAGEN, Venlo, Netherlands), according to the manufacturer’s instructions. Exome sequencing of parental HCT116 and HCT116R^F10^ cells was performed by APRO Life Science Institute, Inc. (Tokushima, Japan) and Macrogen Global Headquarters (Seoul, Korea).

### 4.8. Western Blot Analysis

Western blot analysis was performed as previously described [29,32,33,34]. The antibodies used were rabbit anti-thymidylate synthase (D5B3) monoclonal antibody (9045S, 1:1000, Cell Signaling Technologies, Danvers, MA, USA), mouse anti-DPYD (A-5) monoclonal antibody (sc-376712, 1:1000, Santa Cruz Biotechnology, Dallas, TX, USA), rabbit anti-GAPDH antibody (2275-PC-100, 1:20,000, Trevigen, Gaithersburg, MD, USA), mouse anti-beta-actin monoclonal antibody (A19178-200UL, 1:20,000, Sigma-Aldrich), horseradish peroxidase-linked anti-rabbit IgG (1:20,000, GE Healthcare, Pittsburgh, PA, USA), and horseradish peroxidase-linked whole antibody anti-mouse IgG (1:20,000, GE Healthcare).

### 4.9. Statistical Analysis

The data are presented as means ± standard deviation. The significance of differences among groups was evaluated using a Student’s *t*-test; *p* < 0.05 was considered statistically significant.

## Figures and Tables

**Table 1:** Sensitivity of 5-fluorouracil in the parental HCT116, HCT116R^F3^ and HCT116R^F10^ cells.

Cell line	EC_50_ (M)		RI	
	WST-8 Assay	CFA	WST-8 Assay	CFA
HCT116	5.1 × 10^−6^	5.5 × 10^−6^	1	1
HCT116R^F3^	1.5 × 10^−5^	1.6 × 10^−5^	2.9	2.9
HCT116R^F10^	2.9 × 10^−5^	3.8 × 10^−5^	5.7	6.9

Note. EC_50_, 50% effective concentration; R, resistant; F3, fluorouracil 3 × 10^−6^ M; F10, fluorouracil 10 × 10^−6^ M; RI, resistance index.

**Table 2:** Sensitivities of several anticancer agents in the parental HCT116 and HCT116R^F10^ cells.

	EC_50_ (M, WST-8)			EC_50_ (M, CFA)		
	HCT116	HCT116R^F10^	RI	HCT116	HCT116R^F10^	RI
FUdR	1.5 × 10^−6^	1.2 × 10^−4^	80.0	3.4 × 10^−6^	3.3 × 10^−5^	9.7
SN-38	3.1 × 10^−9^	6.6 × 10^−9^	2.1	4.2 × 10^−9^	3.0 × 10^−9^	0.7
CDDP	1.0 × 10^−5^	1.4 × 10^−5^	1.4	5.2 × 10^−6^	4.5 × 10^−6^	0.9

Note: EC_50_, 50% effective concentration; R^F10^, resistant to fluorouracil 10 × 10^−6^ M; RI, resistance index.

**Table 3:** Mutations of 5-FU metabolic enzyme genes in the parental HCT116 and HCT116R^F10^ cells.

Gene Symbol	HCT116	HCT116R^F10^
***DPYD***	***wt*** *mt*(c.2908-58G>C)*het* *mt*(.2907+55C>T)*hom* ***wt*** ***wt*** ***mt*(c.2442+77_2442+78delAA)*het*** *mt*(c.2059-94G>T)*het* *mt*(c.1740+40A>G)*hom* *mt*(c.1740+39C>T)*het* *mt*(c.1627A>G)*het* *mt*(c.234-123G>C)*het* ***mt*(c.40-461delT)*het*** ***mt*(c.-113T>C)*het***	***mt*(c.2999A>T)*het*** *mt*(c.2908-58G>C)*het* *mt*(.2907+55C>T)*hom* ***mt*(c.2623-59T>G)*het*** ***mt*(c.2442+78delA)*het*** ***wt*** *mt*(c.2059-94G>T)*het* *mt*(c.1740+40A>G)*hom* *mt*(c.1740+39C>T)*het* *mt*(c.1627A>G)*het* *mt*(c.234-123G>C)*het* ***wt*** ***wt***
***DPYS***	*mt*(c.1444-145C>T)*hom* *mt*(c.951-113T>C)*hom* *mt*(c.424-62G>T)*hom* *mt*(c.265-58T>C)*hom* *mt*(c.216C>T)*hom* *mt*(c.-1T>C)*hom*	*mt*(c.1444-145C>T)*hom* *mt*(c.951-113T>C)*hom* *mt*(c.424-62G>T)*hom* *mt*(c.265-58T>C)*hom* *mt*(c.216C>T)*hom* *mt*(c.-1T>C)*hom*
***BUPI***	n.d.	n.d.
***TP***	n.d.	n.d.
***TK1***	***mt*(c.393+168C>T)*het*** ***mt*(c.393+1G>A)*het*** *mt*(c.225A>G)*het* *mt*(c.98+97_98+101delCCCCT)*het* *mt*(c.33T>C)*het*	***wt*** ***wt*** *mt*(c.225A>G)*het* *mt*(c.98+97_98+101delCCCCT)*het* *mt*(c.33T>C)*het*
***TK2***	*mt*(c.619-53A>G)*het* *mt*(c.619-63C>G)*het* *mt*(c.156+836G>A)*het* *mt*(c.156+742G>A)*het* *mt*(c.125-116G>A)*het* *mt*(c.-30C>G)*het* *mt*(c.-38A>G)*het*	*mt*(c.619-53A>G)*het* *mt*(c.619-63C>G)*het* *mt*(c.156+836G>A)*het* *mt*(c.156+742G>A)*het* *mt*(c.125-116G>A)*het* *mt*(c.-30C>G)*het* *mt*(c.-38A>G)*het*
***TYMS***	*mt*(c.97T>C)*het* *mt*(c.280-43G>A)*hom* *mt*(c.454+50T>C)*hom* ***wt*** ***wt*** ***mt*(c.454+200_454+202delTTT)*hom*** *mt*(c.556+123_556+126delATTG)*hom* *mt*(c.*19C>T)*hom* *mt*(c.*89A>G)*het*	*mt*(c.97T>C)*het* *mt*(c.280-43G>A)*hom* *mt*(c.454+50T>C)*hom* ***mt*(c.454+197_454+202delTTTTTT)*het*** ***mt*(c.454+199_454+202delTTTT)*het*** ***wt*** *mt*(c.556+123_556+126delATTG)*hom* *mt*(c.*19C>T)*hom* *mt*(c.*89A>G)*het*
***DHFR1***	***wt*** ***wt*** *mt*(c.-204T>C)*het*	***mt*(c.137-25T>G)*het*** ***mt*(c.137-43T>C)*het*** *mt*(c.-204T>C)*het*
***DHFR2***	*mt*(c.247C>G)*hom*	*mt*(c.247C>G)*hom*
***SHMT1***	*mt*(c.*66C>T)*het* *mt*(c.*47C>G)*het* *mt*(c.1420C>T)*het* *mt*(c.1171+59A>G)*het* *mt*(c.1054+141C>T)*het* *mt*(c.815-23C>T)*het* *mt*(c.601+174C>T)*het* *mt*(c.601+173G>A)*het* *mt*(c.243-256A>G)*het* *mt*(c.-19-101T>C)*hom*	*mt*(c.*66C>T)*het* *mt*(c.*47C>G)*het* *mt*(c.1420C>T)*het* *mt*(c.1171+59A>G)*het* *mt*(c.1054+141C>T)*het* *mt*(c.815-23C>T)*het* *mt*(c.601+174C>T)*het* *mt*(c.601+173G>A)*het* *mt*(c.243-256A>G)*het* *mt*(c.-19-101T>C)*hom*
***SHMT2***	*mt*(c.595-6G>A)*het* *mt*(c.717+14dupG)*het* *mt*(c.1279+30G>A)*het*	*mt*(c.595-6G>A)*het* *mt*(c.717+14dupG)*het* *mt*(c.1279+30G>A)*het*

Note. *wt*, wild-type; *mt*, mutation-type; n.d., not detected; *hom*, homozygous; *het*, heterozygous.

## Data Availability

Not applicable.
